# Occurrence of *Culex* (*Microculex*) Theobald, 1907 in a polyvinyl chloride trap container indicates an ongoing adaptation process to anthropized environment

**DOI:** 10.1590/S1678-9946202668053

**Published:** 2026-08-07

**Authors:** Rafael Oliveira-Christe, Luiz Carlos de Oliveira, Jair Donizete da Silva, Mauro Toledo Marrelli

**Affiliations:** 1 Universidade de São Paulo, Faculdade de Saúde Pública, Departamento de Epidemiologia, São Paulo, São Paulo, Brazil

**Keywords:** Mosquitoes, Ecology, Artificial container.

## Abstract

Microculex mosquitoes are widespread in Central and South America and typically oviposit in phytotelmata breeding sites. However, some species also tend to oviposit in artificial containers. Artificial containers made of polyvinyl chloride (PVC traps) were installed and monitored in Pariquera-Acu and Cananeia, São Paulo State, Brazil for six months (December 2024 to August 2025) to test their efficiencies in collecting mosquitoes. Of the four collected species, three are considered sylvatic species: *Toxorhynchites* (*Megarhina*) *theobaldi*, *Culex* (*Microculex*) *imitator*, and *Limatus durhamii*. *Culex* (*Microculex*) *imitator* specimens were collected for the first time in an artificial breeding site. This finding indicates that *Cx. imitator* tend to use artificial containers for reproduction. Continuous monitoring and research on the ecology and epidemiological relevance of *Cx. imitator* is essential given the limited information on its potential role as an arbovirus vector.

## INTRODUCTION

*Culex* (*Microculex*) Theobald, 1903, is a subgenus of mosquitoes within the genus *Culex* Linnaeus, 1758, comprising 34 described species characterized by their occurrence in phytotelmata breeding sites, such as bromeliads, bamboo internodes, and tree holes^1^. However, some of these species, such as *Cx.* (*Microculex*) pleuristriatus Theobald, 1903, tend to oviposit in artificial breeding sites, including tires and plastic containers^2^. Some species within this subgenus have also adapted to reproduce in bromeliads in areas affected by anthropogenic impacts and loss of vegetation cover^3^. 

The adaptation of sylvatic native Culicidae species to artificial breeding sites holds epidemiological importance due to the role of these insects as disease vectors^4^. Changes in ecological and/or biological factors may lead potential vectors to exploit new environments and feed on different hosts^5^. Oria *et al*.^6^ found *Cx* (*Mcx.*) *imitator*, Theobald, 1903, infected with Sant Louis encephalitis virus at Pampa del Indio, Argentina^6^. This study proposes a six-month field experiment (summer and autumn) to investigate the potential of sylvatic mosquito species native to the Atlantic Forest to oviposit in artificial traps made of polyvinyl chloride (PVC) and to discuss the ecological and epidemiological implications of this behavior.

## MATERIALS AND METHODS

**Figure 1:**
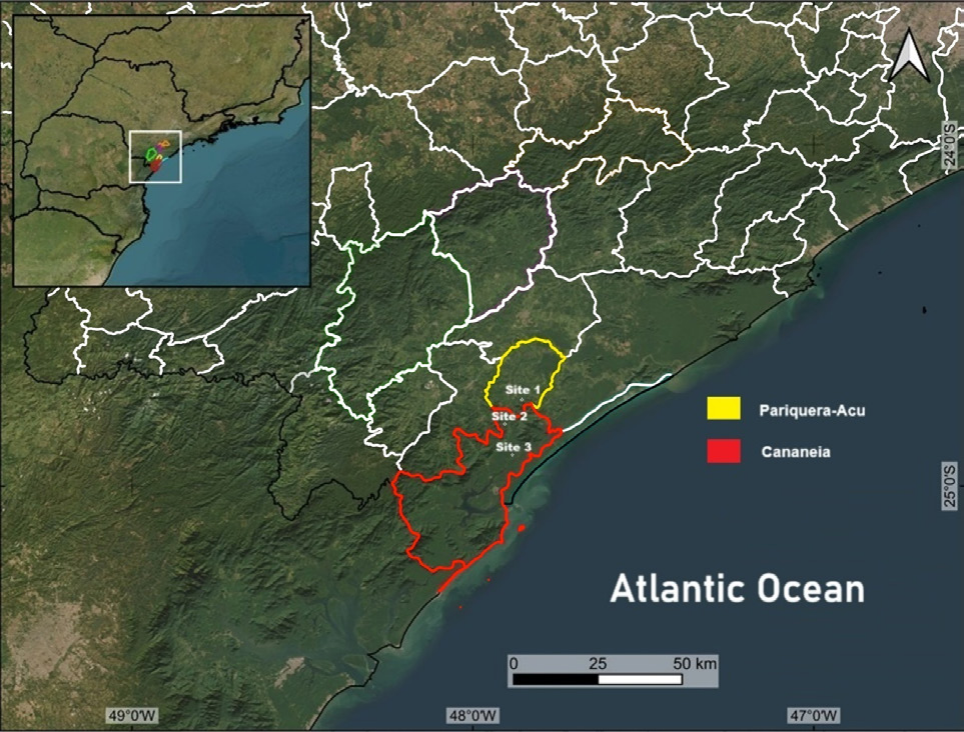
Locations of the collection sites in Pariquera Acu and Cananeia, Sao Paulo State, Brazil.

**Figure 2:**
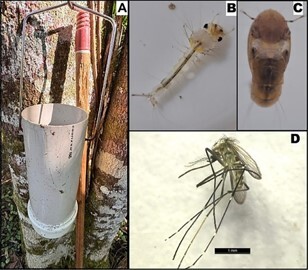
A) PVC trap; B) *Culex* (*Microculex*) *imitator* larvae; C) *Culex* (*Microculex*) *imitator* pupae; D) *Culex* (*Microculex*) *imitator* adult (female).

In total, three sites in the Atlantic Forest of Sao Paulo State, Brazil, were selected in the municipalities of Pariquera-Acu and Cananeia: Site A (24°44’43.2”S, 47°51’02.9”W), B (24°51’59.2”S, 47°52’42.2”W), and C (24°28’22.4”S, 47°32’39.3”W). Artificial containers were installed in these sites (Figure 1)-situated in preserved and transitional areas. These containers (Figure 2A) consisted of white PVC cylindrical tubes with a 200-mm diameter. These tubes were 40 cm long, and their bases were sealed with caps of the same diameter. They had open tops. A steel handle was attached near the upper edge of each trap four meters above the ground to facilitate installation and removal. At each collection site, one PVC trap with about 400 mL of dechlorinated water was installed three meters from the ground. When evaporation significantly reduced the water level (below 200ml), the volume was replenished with the same type of water. These containers were maintained in the field for six months (from December 2024 to August 2025). Once per week, the presence of Culicidae larvae was inspected, and any collected larvae and pupae were transported to the Public Health Entomology Laboratory (LESP/USP), where they were reared until adult emergence. The specimen-rearing procedures included (i) separating each larva and maintaining it individually in a 20-mL plastic container labeled with field collection information (date and site); (ii) feeding the larvae with macerated TetraMin^®^ fish food (Tetra Spectrum Brands, Blacksburg, VA) (after the larvae metamorphosized into pupae, the exuviae were transferred to 1.5-mL microtubes containing 70% ethanol); (iii) covering each pupae-holding container with a protective mesh to prevent the escape of emerging adults; (iv) euthanizing specimens after adult emergence using ethyl acetate and transferring the pupal exuviae to the same microtube containing the larval exuviae; (v) identifying adult specimens by a taxonomic key^7^, individually storing them in 1.5-mL microtubes, associating each adult specimen with the corresponding larval and pupal exuviae via a unique internal code, and storing them in a −20 °C freezer for future analyses; and (vi) removing and dissecting the genitalia of male specimens and mounting them on slides using natural Canada balsam for microscopic examination. Identification of male genitalia was performed using a specific taxonomic key^8^.

## RESULTS AND DISCUSSION

The experimental period collected larvae of four Culicidae species in the PVC traps: *Toxorhynchites* (*Megarhina*) *theobaldi*, (Dyar & Knabi, 1906); *Culex* (*Microculex*) *imitator*, *Culex* (*Culex*) *quinquefasciatus* Say, 1823; and *Limatus durhamii* Theobald, 1901 (Table 1). Previous studies have reported *Tx*. *theobaldi* in artificial containers^9^. Of the *Toxorhynchites* species, the only genus in the tribe Toxorhynchitini-*Tx*. *theobaldi*-is not the only species that can breed in artificial containers. Studies have also reported the occurrence of *Li. durhamii* in artificial containers (tires, plastic, glass, and metal containers)^10^ ^-^ ^12^. This phenomenon may be attributed to ecological adaptations that enable these sylvatic mosquitoes to exploit breeding sites densely occupied by other species, such as *Aedes aegypti* (Linnaeus, 1792) and *Aedes albopictus* (Skuse, 1894)^13^. This atypical behavior for sylvatic mosquitoes, combined with the predatory habit of their larvae, has led some researchers to propose the use of this species as a biological control strategy to suppress vector mosquito populations in urbanized or transitional areas. 

**Table 1:** Diversity of mosquitoes collected from December 2024 to August 2025, in PVC traps at Pariquera-Acu and Cananeia, Sao Paulo State, Brazil

Species	Site A	Site B	Site C	Period
*Culex* (*Microculex) imitator*	3		1	January 2025
*Limatus durhamii*	2			March 2025
*Culex* (*Culex*) *quinquefasciatus*	1			March 2025
*Toxorhynchites* (*Megarhina*) *theobald*	1			March 2025
*Culex* (*Microculex) imitator*	1			April 2025
*Culex* (*Microculex) imitator*	1			June 2025
*Culex* (*Microculex) imitator*	1			August 2025
**Total**	**10**	**0**	**1**	

This study found *Culex* (*Microculex*) *imitator* breeding in artificial containers for the first time (Figures 2B to 2D). It collected *Cx*. *imitator* specimens on multiple sampling occasions, providing evidence of their tolerance to artificial environments and excluding accidental occurrence. Their repeated presence in the PVC trap at Site A may indicate that impacted environments can drive native mosquito species to exploit artificial breeding sites, as reported for *Li. Durhamii* ^12^. In general, the subgenus *Culex* (*Microculex*) comprises sylvatic species commonly found in tree holes and, more frequently, in bromeliads in wild and transitional zones^14^. Dorvillé^15^ considers the subgenus *Microculex* as a bioindicator of preserved areas. However, some studies suggest that certain species show ecological flexibility, exploiting bromeliads in impacted environments^14^. Oliveira-Christe *et al.* ^1^ have described the ecological profiles of 14*Culex Microculex* species, some of which-such as *Cx*. (*Mcx*.) *aphylactus* Root, 1927, *Cx*. (*Mcx*.) *microphyllus* Root, 1927, and *Cx*. (*Mcx*.) *inimitabilis* Dyar & Knab, 1906-occur more often in areas with dense forest cover, whereas others, including Cx. imitator and Cx. pleuristriatus, occur more abundantly in areas with reduced forest cover. Notably, *Cx*. *pleuristriatus*, *Cx*. (*Mcx*.) *albipes* Lutz, 1904, and *Cx*. (*Mcx*.) *aureus* Lane & Whitman, 1951, (all belonging to the Pleuristriatus series) constituted the only larval species found in artificial containers. 

## CONCLUSION

A study on blood-feeding habits of the subgenus *Microculex* suggests their tendency to feed on amphibians^16^. Alencar *et al*.^17^ have shown that can feed on birds and rodents, whereas Santos *et al*.^18^ reported that *Cx. imitator* feeds on amphibians and birds. The occurrence of *Cx. imitator* in artificial breeding sites indicates an ongoing adaptation process, possibly driven by environmental pressures such as the reduction of bromeliads in the area. The ability to exploit new oviposition sites may also change blood-feeding behavior, holding potential epidemiological implications. 

## Data Availability

The complete anonymized dataset supporting the findings of this study is included within the article itself.
